# Effect of gallic acid on the larvae of *Spodoptera litura* and its parasitoid *Bracon hebetor*

**DOI:** 10.1038/s41598-020-80232-1

**Published:** 2021-01-12

**Authors:** Abhay Punia, Nalini Singh Chauhan, Drishtant Singh, Anup Kumar Kesavan, Sanehdeep Kaur, Satwinder Kaur Sohal

**Affiliations:** Department of Zoology, Guru Nanak Dev University, Amritsar, Punjab India

**Keywords:** Ecology, Plant sciences, Zoology

## Abstract

The antibiosis effect of gallic acid on *Spodoptera litura* F. (Lepidoptera: Noctuidae) and its parasitoid evaluated by feeding six days old larvae on artificial diet incorporated with different concentrations (5 ppm, 25 ppm, 125 ppm, 625 ppm, 3125 ppm) of the phenolic compound revealed higher concentration (LC_50_) of gallic acid had a negative impact on the survival and physiology of *S. litura* and its parasitoid *Bracon hebetor* (Say) (Hymenoptera:Braconidae). The mortality of *S. litura* larvae was increased whereas adult emergence declined with increasing concentration of gallic acid. The developmental period was delayed significantly and all the nutritional indices were reduced significantly with increase in concentration. Higher concentration (LC_50_) of gallic acid adversely affected egg hatching, larval mortality, adult emergence and total development period of *B. hebetor.* At lower concentration (LC_30_) the effect on *B. hebetor* adults and larvae was non-significant with respect to control. Gene expression for the enzymes viz*.,* Superoxide dismutase, Glutathione peroxidase, Peroxidase, Esterases and Glutathione S transferases increased while the total hemocyte count of *S. litura* larvae decreased with treatment. Our findings suggest that gallic acid even at lower concentration (LC_30_) can impair the growth of *S. litura* larvae without causing any significant harm to its parasitoid *B. hebetor* and has immense potential to be used as biopesticides.

## Introduction

Parasitoids have a very close association with the insect pests and their host plants. They are natural biological control agents which can regulate the pest population. The pest populations are also kept in check by plant phytochemicals, a majority of which evolved as defensive compounds which deter herbivory. These chemicals commonly known as secondary metabolites or allelochemicals regulate pest population by imparting toxic or feeding deterrent activity in insect pests^1^.Variations in these compounds can also affect the fitness of natural enemies of insect pests.

Phenolics are plant secondary metabolites widely distributed in plants, and are helpful to them in a number of ways such as by providing physical barriers as cell wall bound phenolics, lignin suberin, and cuticle associated phenolics as well as stored compounds that have deterring and insecticidal effect on herbivores^2^. These are reported to be potential sources of new natural drugs, antibiotics, insecticides and herbicides^3,4^. Induced defense response in plants against herbivore attacks lead to an increase in the production of foliage phenolic content^5^. Phenolic acids are a diverse class of phenols in plants and are also known as hydrobenzoates, principle component of which is gallic acid. The formation of gallic acid, a base unit of gallotanins, occurs mainly via the shikimic acid pathway from 3-dehydroxyshikimic acid. However, it can also be formed through alternative routes from hydroxybenzoic acids. Galloylation of gallic acid also results in the synthesis of the hydrolysable tannins viz*.,* gallotannins and ellagitannins^6^. Plant allelochemicals play a key role in the survival of herbivore and are lately being extensively explored as potential alternative to synthetic pesticides. However, plant alleleochemicals in the host diet can not only affect the fitness and survival of the phytophagous insect but can also impact the growth, development and survival of parasitoid because the quality of the host for its parasitoid^7,8^ is determined by the quality of the host's nutrient intake^9^. Several studies have also reported that secondary compounds ingested by the host can negatively affect parasitoids^10–13^. Therefore before incorporating these compounds in pest management programmes, it is essential to study their effect not only on the insect pest but also on its parasitoids as the latter play a key role in limiting insect pest population.

*S. litura* is an indigenous pest of a variety of crops in South Asia and was found to cause 26–100% yield loss in groundnut^14^. Mainly due to development of insecticide resistance, its frequent outbreaks have become more common^15,16^. *B. hebetor*, a gregarious ectoparasitoid attacking some storage moths^17^, has been intensively studied because of its importance as a biological control agent of the moths^18,19^. Biology of *B. hebetor* varies with the host age, nutritional status and freshness of the host^20^. The present study was therefore aimed at investigating the influence of gallic acid on the common cutworm, *Spodoptera litura* (F.) and its parasitoid, *Bracon hebetor* (Say).

## Results

### Effect on growth and development of *S. litura* larvae

Gallic acid incorporated diet when fed to the six days old larvae of *S. litura* significantly affected the growth, development and survival of *S. litura* larvae (Table 1). In gallic acid treated *S. litura* larvae, the larval mortality significantly increased in a dose dependent manner from 3.33% in control to 70% at 3125 ppm. The percentage adult emergence decreased with increase in concentration. No adult emerged at the highest concentration of 3125 ppm. At 625 ppm the adult emergence was inhibited by 73.33% when compared with control. The larval period and total developmental period were delayed significantly after feeding larvae with diet having gallic acid when compared with control. The larval period was delayed by 13.52 days at 3125 ppm concentration while delay of 10.37 days in total developmental period was observed at 625 ppm as compared to control. The pupal weight was also significantly lower at all the concentrations when compared with control. The LC_30_ and LC_50_ calculated on the basis of larval mortality were 52.44 ppm and 402.80 ppm concentrations, respectively. Growth and survival of azadirachtin treated positive control *S. litura* larvae was more adversely affected when compared to gallic acid fed larvae. At higher concentration of 3125 ppm, 93.33% larval mortality was observed in azadirachtin fed larvae (Table 2). The larval period and total developmental period were also more prolonged in azadirachtin treated larvae. The LC_30_ and LC_50_ of azadirachtin fed larvae was 5.981 ppm and 26.551 ppm, respectively.

**Table 1:** Larval mortality, adult emergence, larval period, pupal period, total developmental period, pupal weight of *S. litura* when 6 days old instar larvae were fed on different concentrations of gallic acid.

Concentrations (ppm)	Larval mortality (%)	Adult emergence (%)	Larval period (days)	Pupal period (days)	Total development period (days)	Pupal weight (mg)
Control	3.33 ± 3.33^a^	90.00 ± 4.47^a^	19.39 ± 0.47^a^	13.56 ± 0.59	33.38 ± 0.88^a^	254.16 ± 6.84^a^
5	6.67 ± 4.22^a^	80.00 ± 5.16^a^	20.96 ± 0.49^ad^	14.46 ± 0.58	35.30 ± 0.40^ab^	253.78 ± 7.01^a^
25	30.00 ± 8.56^ab^	50.00 ± 6.83^b^	22.58 ± 0.53^d^	14.39 ± 0.57	37.45 ± 1.09^b^	231.37 ± 9.51^a^
125	46.67 ± 4.22^bc^	26.67 ± 6.67^c^	30.92 ± 0.32^b^	14.79 ± 0.72	43.74 ± 0.73^c^	155.87 ± 7.08^b^
625	46.70 ± 11.20^bc^	16.67 ± 6.15^ cd^	29.00 ± 0.30^c^	16.00 ± 0.57	43.75 ± 0.83^c^	174.43 ± 9.70^b^
3125	70.00 ± 13.40^c^	0.00 ± 0.00^d^	32.91 ± 0.22^e^	__	^__^	156.22 ± 4.34^b^
F-value	9.39**	43.96**	193.54**	ns	42.82**	38.53**

**Significant at 1%, ^ns^Non-Significant. Values are Mean ± SE. Means followed by the same letter within the columns are not significantly different according to Tukey’s test at *p* ≤ 0.05.

**Table 2:** Larval mortality, adult emergence, larval period, pupal period, total developmental period, pupal weight of *S. litura* when 6 days old instar larvae were fed on different concentrations of azadirachtin.

Concentrations (ppm)	Larval mortality (%)	Adult emergence (%)	Larval period (days)	Pupal period (days)	Total development period (days)	Pupal weight (mg)
Control	3.33 ± 3.33^a^	90.00 ± 4.47^a^	96.68 ± 0.27^a^	13.62 ± 0.58^a^	33.17 ± 0.74^a^	254.16 ± 6.84^a^
5	26.67 ± 4.21^b^	46.67 ± 4.21^b^	22.39 ± 0.60^b^	15.33 ± 0.21^ab^	37.72 ± 0.71^b^	231.12 ± 2.97^ab^
25	50.00 ± 4.40^c^	30.00 ± 4.47^c^	27.92 ± 0.32^c^	15.94 ± 0.34^bc^	43.78 ± 0.54^c^	214.80 ± 5.33^b^
125	70.00 ± 4.47^d^	10.00 ± 4.47^d^	29.00 ± 0.50^c^	16.67 ± 0.21^bcd^	45.67 ± 0.57^c^	155.76 ± 7.04^c^
625	90.00 ± 4.47e	3.33 ± 3.33^d^	35.33 ± 0.33^d^	17.67 ± 0.33^ cd^	52.67 ± 0.33^d^	135.17 ± 2.89^c^
3125	93.33 ± 4.21^e^	0.00 ± 0.00^d^	36.00 ± 0.00^d^	____	^____^	110 ± 0.00^d^
F-value	119.00**	79.50**	160.11**	14.86**	108.41**	62.59**

**Significant at 1%. Values are Mean ± SE. Means followed by the same letter within the columns are not significantly different according to Tukey’s test at *p* ≤ 0.05.

### Effect on the nutritional physiology of *S. litura*

The nutritional physiology of the larvae was also negatively affected in gallic acid treated larvae as evident from observations recorded for various nutritional indices (Table 3). The AD (approximate digestibility) of the larvae declined with treatment in comparison to control. The ECD (efficiency of conversion of digested food) and ECI (efficiency of conversion of ingested food) showed a significant concentration dependent decline. At the highest concentration of 3125 ppm the ECD decreased by 56.69% and ECI decreased by 64.16% respectively when compared with control. The RGR (relative growth rate) and RCR (relative consumption rate) also decreased significantly in the larvae of *S. litura* with increasing concentration of gallic acid incorporated in diet. At 3125 ppm, a 87.88% reduction in RGR and 65.37% reduction in RCR was noticed as compared to control. The nutritional physiology of the larvae was more adversely affected when fed on azadirachtin treated diet when compared to control and gallic acid treated larvae (Table 4).

**Table 3:** Nutritional indices of *S. litura* when 6 days old instar larvae were fed on different concentrations of gallic acid.

Concentrations (ppm)	RGR (mg/mg/day)	RCR (mg/mg/day)	ECI (%)	ECD (%)	AD (%)
Control	1.67 ± 0.09^a^	18.05 ± 0.80^a^	9.31 ± 0.47^a^	10.84 ± 0.67^a^	86.89 ± 1.24^a^
5	0.92 ± 0.06^b^	13.79 ± 0.64^b^	6.85 ± 0.32^b^	8.32 ± 0.38^b^	82.99 ± 2.27^ab^
25	0.70 ± 0.04^bc^	11.98 ± 0.79^bc^	6.02 ± 0.43^bc^	7.54 ± 0.51^bc^	80.51 ± 2.28^ab^
125	0.51 ± 0.03^ cd^	10.67 ± 0.56^c^	4.66 ± 0.25^ cd^	6.58 ± 0.44^bcd^	72.46 ± 3.03^b^
625	0.36 ± 0.03^de^	8.07 ± 0.27^d^	4.62 ± 0.42^ cd^	6.11 ± 0.48^ cd^	78.26 ± 4.02^ab^
3125	0.20 ± 0.02^e^	6.26 ± 0.20^d^	3.34 ± 0.69^d^	4.69 ± 0.39^d^	77.27 ± 2.43^ab^
F-value	102.53**	50.35**	32.47**	18.75**	3.69*

**Significant at 1%, *Significant at 5%. Values are Mean ± SE. Means followed by the same letter within the columns are not significantly different according to Tukey’s test at *p* ≤ 0.05.

**Table 4:** Nutritional indices of *S. litura* when 6 days old instar larvae were fed on different concentrations of azadirachtin.

Concentrations (ppm)	RGR (mg/mg/day)	RCR (mg/mg/day)	ECI (%)	ECD (%)	AD (%)
Control	1.67 ± 0.09^a^	18.05 ± 0.80^a^	9.31 ± 0.47^a^	10.84 ± 0.70^a^	86.89 ± 1.24^a^
5	0.81 ± 0.04^b^	8.33 ± 0.18^b^	6.61 ± 0.16^b^	7.50 ± 0.32^b^	72.84 ± 2.07^b^
25	0.58 ± 0.01^c^	7.74 ± 0.11^b^	5.10 ± 0.23^c^	6.21 ± 0.31^bc^	70.46 ± 0.71^b^
125	0.38 ± 0.02^a^	7.14 ± 0.20^b^	3.92 ± 0.21^d^	4.47 ± 0.40^ cd^	65.36 ± 1.41^bc^
625	0.17 ± 0.01^e^	5.21 ± 0.30^c^	2.20 ± 0.25^e^	3.90 ± 0.28^d^	60.38 ± 3.45^ cd^
3125	0.10 ± 0.01^e^	4.27 ± 0.35^c^	1.86 ± 0.19^e^	3.30 ± 0.38^d^	56.20 ± 2.59^d^
F-value	172.97**	186.37**	107.80**	46.02**	26.12**

**Significant at 1%. Values are Mean ± SE. Means followed by the same letter within the columns are not significantly different according to Tukey’s test at *p* ≤ 0.05.

### Effect of gallic acid on the cellular immune response of *S. litura*

**Figure 1:**
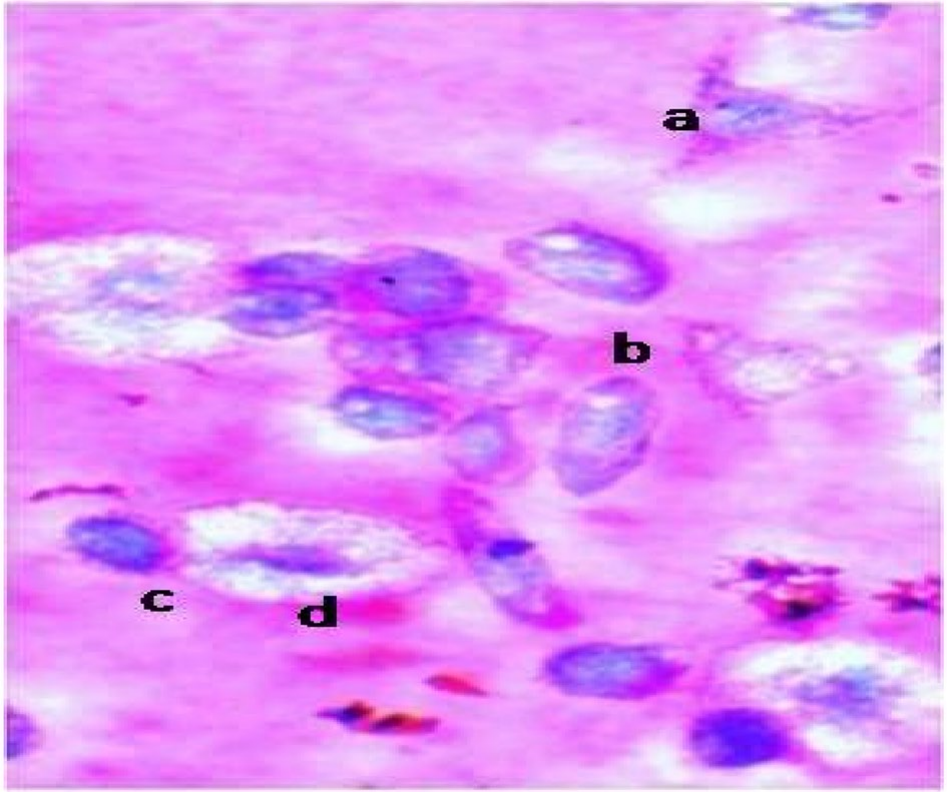
(a, b) Normal plasmatocytes (c) Normal granulocyte (d) Normal spherulocyte.

**Figure 2:**
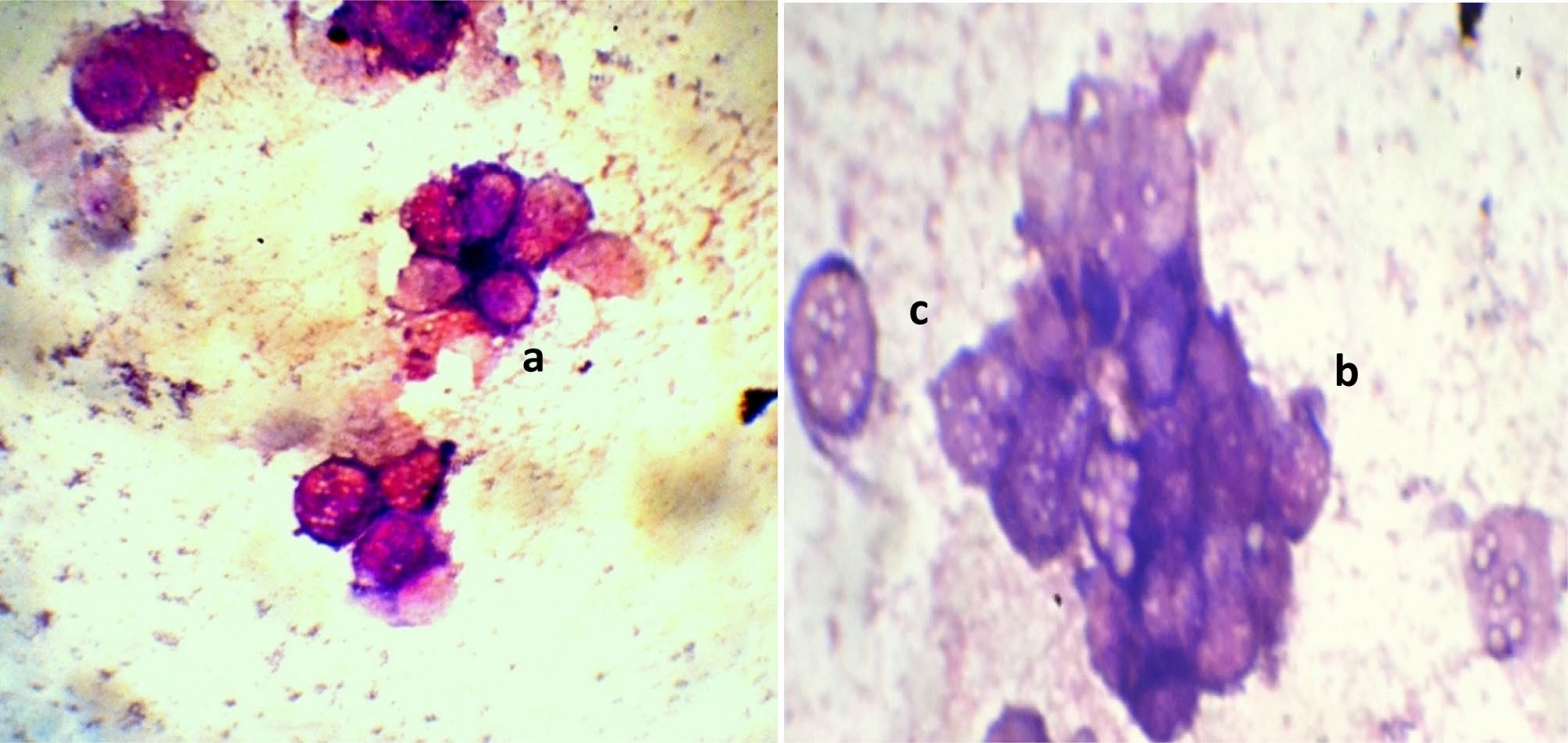
(a) Necrosis (b) Clustering (c) Vacuolization of hemocytes.

At all treatment intervals the percent hemocyte deformity increased significantly and was maximum in larvae fed on LC_50_ concentration when compared with control (Table 5). Hemocytes deformities were observed in the form of clustering, necrosis and vacuolization of hemocytes (Figs. 1, 2). The total haemocyte count in larvae fed on diet amended with LC_50_ and LC_30_ concentration of the gallic acid also significantly declined at all the treatment intervals when compared with control (Table 6).

**Table 5:** Hemocyte deformities (%) in *S. litura* larvae under the influence of gallic acid.

Concentrations (ppm)	24 h	48 h	72 h	96 h	120 h
Control	1.25 ± 0.11^a^	1.83 ± 0.26^a^	1.67 ± 0.10^a^	1.92 ± 0.15^a^	2.58 ± 0.24^a^
LC_30_ (52.44)	7.17 ± 0.28^b^	8.25 ± 0.28^b^	13.08 ± 0.86^b^	15.58 ± 0.51^b^	23.67 ± 0.28^b^
LC_50_ (402.80)	14.58 ± 0.20^c^	19.67 ± 0.40^c^	24.58 ± 1.59^c^	29.50 ± 0.48^c^	45.25 ± 0.44^c^
F-value	1025.59**	973.67**	705.46**	1110.45**	4130.61**

**Significant at 1%. Values are Mean ± SE. Means followed by different superscript letters within a column are significantly different (*p* ≤ 0.05) based on Tukey’s test.

**Table 6:** Effect of gallic acid on total hemocyte count (Total number of hemocytes/mm^3^) of *S. litura* larvae.

Concentrations (ppm)	24 h	48 h	72 h	96 h	120 h
Control	9533.3 ± 84.30^a^	9766.7 ± 61.50^a^	10,467 ± 84.30^a^	11,267 ± 66.70^a^	11,400 ± 61.70^a^
LC_30_ (52.44)	9166.7 ± 61.50^b^	8366.7 ± 80.30^b^	8266.7 ± 42.20^b^	7633 ± 120.00^b^	7533 ± 66.70^b^
LC_50_ (402.80)	8733.3 ± 66.70^c^	7400.0 ± 51.60^c^	7266.7 ± 42.20^c^	7000 ± 89.40^c^	6766.7 ± 61.50^c^
F-value	31.38**	329.57**	753.75**	591.45**	1365**

**Significant at 1%. Values are Mean ± SE. Means followed by different superscript letters within a column are significantly different (*p* ≤ 0.05) based on Tukey’s test.

### Quantitative real time PCR (qrt-PCR) assay on *S. litura* larvae

**Figure 3:**
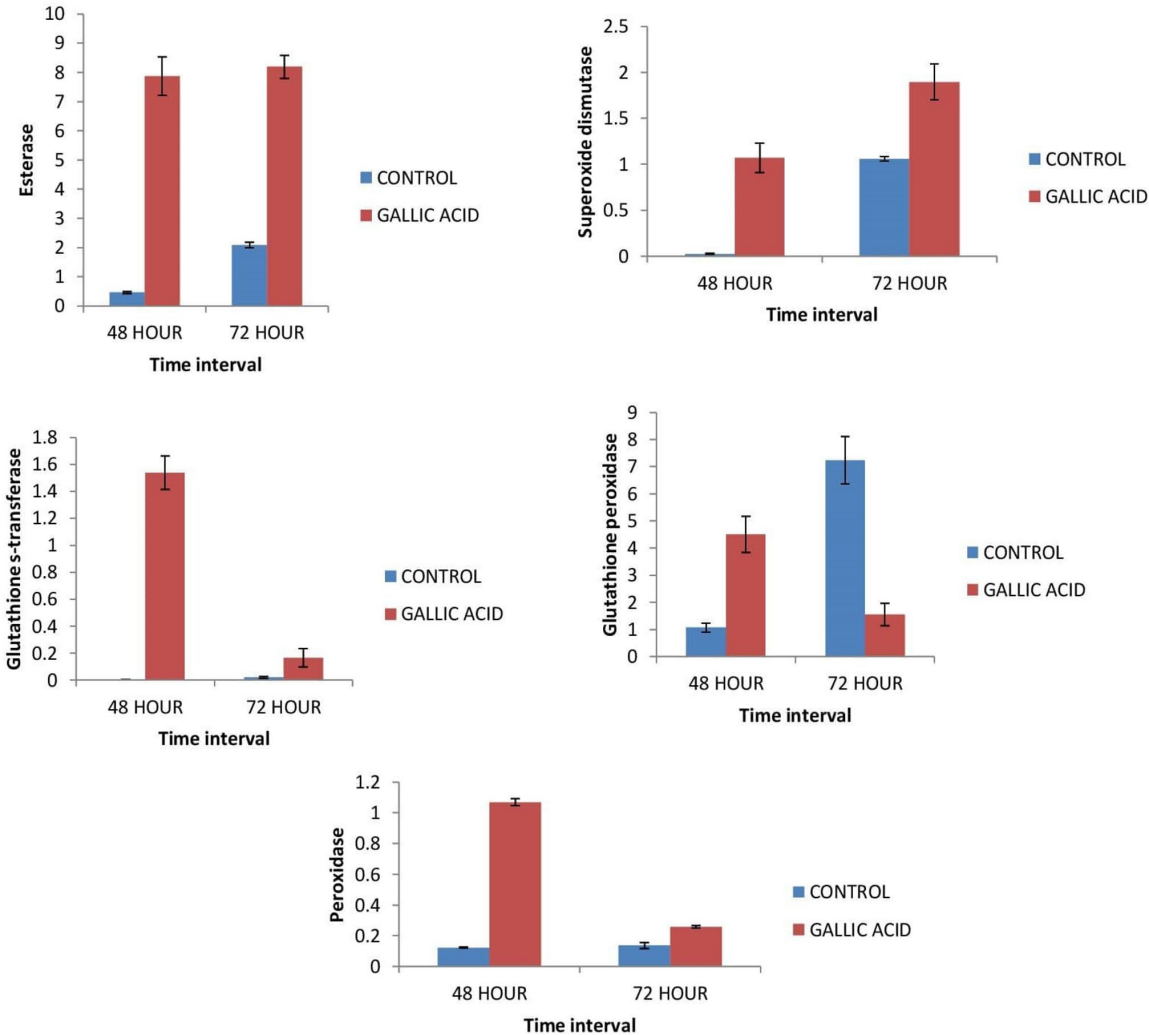
Effect on the expression (Means ± S.E) of different antioxidant genes (EST, SOD, GST, GPOX and POX) of *S. litura* larvae treated with artificial diet amended with and without gallic acid.

Gene expression of Superoxide Dismutase (SOD), Esterase (EST), GST (Glutathione S- transferase), Glutathione peroxidase (GPOX) and Peroxidase (POX) increased significantly in 3rd instar larvae of *S. litura* fed on gallic acid at 48 and 72 h treatment intervals with respect to control. Esterase, SOD, GST and POX expression showed maximum increase at 48 h interval. Gene expression of Glutathione peroxidase at 48 h interval showed a 4.23 fold increase as compared to control but declined at 72 h interval (Fig. 3).

### Effect on *B. hebetor* adults

Gallic acid showed no significant effect on female fecundity, percent parasitization and the eggs laid per host at both LC_50_ and LC_30_ concentrations when compared to control (Table 7).

**Table 7:** Parasitization, no. of parasitized egg/host and fecundity of *B. hebetor* adults reared on *S. litura* larvae fed on gallic acid and azadirachtin supplemented diet.

Concentrations (ppm)	Parasitization (%)	Eggs per host	Fecundity (%)
Control	80.00 ± 7.75^a^	12.12 ± 0.72^a^	45.83 ± 4.26^a^
(GA) LC_30_ (52.44)	76.76 ± 2.71^a^	11.67 ± 2.33^a^	36.75 ± 9.41^a^
(GA)LC_50_ (402.80)	60.00 ± 6.83^a^	9.16 ± 0.77^a^	30.33 ± 3.87^a^
(AZA) LC_30_ (5.981)	25.00 ± 2.24^b^	4.18 ± 0.25^b^	8.25 ± 1.03^b^
(AZA) LC_50_ (26.551)	15.00 ± 2.22^b^	1.62 ± 0.26^b^	4.42 ± 0.51^b^
F-value	36.67**	16.29**	13.25**

**Significant at 1%. Values are Mean ± SE. Means followed by the same letter within the columns are not significantly different according to Tukey’s test at *p* ≤ 0.05. GA (Gallic acid), AZA (Azadirachtin).

### Effect on the growth and development of *B. hebetor*

The percent hatching and percent adult emergence of *B. hebetor* larvae declined significantly on *S. litura* larvae treated with LC_50_ and LC_30_ concentration of the phenolic acid. The decline in hatching and adult emergence was noticeably greater at LC_50_ then at LC_30._ A 23% reduction in hatching was observed at LC_50_ whereas only 10% decrease in hatching at LC_30_ concentration was observed when compared to control but increase in hatching and adult emergence was observed when compared with azadirachtin treated larvae. Similarly, the adult emergence was reduced to 34.33% at LC_50_ but at lower concentration of LC_30,_ it was reduced by only 13.52% as compared to control. The inhibition in female emergence was also greater at LC_50_ than at LC_30_ but when compared to positive control an increase in emergence was observed. The larval period and the pupal period were not significantly affected at both the concentrations when compared with control but significant increase in total development period was observed with increase in concentration from LC_30_ to LC_50_ concentration of gallic acid provided to the host with respect to control. Azadirachtin treated *S. ltura* larvae had more significant adverse affect on growth and survival of *B. hebetor* at both LC_30_ and LC_50_ when compared with gallic acid fed larvae. Azadirachtin was found to have more toxic effect at third trophic level (Table 8).

**Table 8:** Development and survival of *B. hebetor* larvae emerged from *S. litura* larvae fed on gallic acid and azadirachtin supplemented diet.

Concentrations (ppm)	Hatching (%)	Larval mortality (%)	Larval period (days)	Cocoon period (days)	Total development period (days)	Adult emergence (%)	Female emergence (%)
Control	92.85 ± 0.91^a^	11.34 ± 1.99^a^	4.70 ± 0.07^a^	5.89 ± 0.07^a^	11.49 ± 0.09^a^	92.93 ± 1.58^a^	62.14 ± 4.40^a^
(GA) LC_30_ (52.44)	83.37 ± 3.01^ab^	17.83 ± 4.38^ab^	4.71 ± 0.16^a^	5.91 ± 0.30^a^	11.63 ± 0.16^a^	80.37 ± 3.68^a^	56.31 ± 4.95^ab^
(GA)LC_50_ (402.80)	71.49 ± 5.77 ^b^	30.48 ± 3.55^b^	4.92 ± 0.05^a^	6.40 ± 0.09^a^	12.70 ± 0.09^b^	61.01 ± 4.35^b^	36.53 ± 7.87^bc^
(AZA) LC_30_ (5.981)	39.92 ± 2.08^c^	49.58 ± 2.11^c^	6.33 ± 0.21^b^	7.50 ± 0.22^b^	14.04 ± 0.26^c^	39.84 ± 3.42^c^	36.62 ± 4.26^bc^
(AZA) LC_50_ (26.551)	27.27 ± 2.16^c^	72.70 ± 1.80^d^	7.00 ± 0.36^b^	7.96 ± 0.26^b^	15.71 ± 0.19^d^	29.30 ± 1.78^c^	20.19 ± 2.56^c^
F-value	76.43**	71.94**	27.31**	19.86**	107.07**	74.40**	10.96**

**Significant at 1%, *Significant at 5%, ^ns^Non significant. Values are Mean ± SE. Means followed by the same letter within the columns are not significantly different according to Tukey’s test at *p* ≤ 0.05.GA (Gallic acid), AZA (Azadirachtin).

## Discussion

The artificial diet containing gallic acid when fed to 6 days old *S. litura* larvae had negative effects on the larvae as well as on its parasitoid, *B. hebetor*. The larval mortality increased and adult emergence decreased under the influence of gallic acid in a concentration dependent manner. Selim-Rani et al.^21^ had also reported that quercetin isolated from *Euphorbia hirta* L. caused mortality and cellular deformities in second, fourth and fifth instar larvae of *S. litura*. Ghumare and Mukherjee^22^ had reported that *S. litura* larvae develop better on plants having lower phenolic content. Ananthakrishnan et al.^23^ had also reported the excessive defecation along with formation of muscular lesions in the posterior half of the *S. litura* larvae after consumption of gallic acid supplemented diet. Prolonged larval and total developmental period of *S. litura* larvae was also noticed. Inability of *S. litura* larvae to develop on artificial diet containing phenolic rich extracts of red gram (*Cajanus cajan* L.) and *Acacia nilotica* (L.) has been reported by Bhattacharya and Chenchiah^24^ and by Gautam et al.^25^, respectively. Gallic acid, hydrolysable tannin can interact with metal ions and macromolecules such as polysaccharides. It can also form soluble complexes with proteins and can inhibit digestive enzymes and is thus toxic to insects^26^.

The nutritional indices were reduced significantly with increase in concentration of gallic acid. Ingestion of gallic acid incorporated diet decreased with a consistent decrease in RGR of *S. litura* larvae. Gallic acid seems to have inhibited growth by either exerting an antifeedant or toxic effect on the *S. litura* larvae. Both ECI and ECD declined with increase in concentration of gallic acid. The decrease in ECD and ECI of *S. litura* larvae reflect increased metabolic cost which might have occurred due to the energy required for detoxification of the compound. These findings indicate toxicity of gallic acid to *S. litura* larvae was due to the inhibitory effect on digestion and reduced efficiency of conversion of assimilated food into biomass. Toxic effects of gallic acid have also been reported against other insects such as melon fruit fly, *Bactrocera cucurbitae* (Coquillett) larvae^27^. Our findings indicated that azadirachtin incorporated diet was more toxic to *S. litura* as compared to gallic acid incorporated diet. The toxic effects of azadirachtin on feeding and mortality of *S. litura* was also reported by Nathan and Kalaivani^28^ and Deota and Upadhyay^29^. Azadirachtin induced structural changes in the *S. litura* larval midgut by activation of apoptosis^30^.

Studies on tritrophic interactions involving plants, insects and their parasitoids have made significant contribution towards understanding the role of plant traits such as induced volatiles on host location and acceptance behavior by natural insect enemies^31^. However, reports linking plant allelochemicals and parasitoids are very few. Most of these reports have highlighted the adverse effects of plant allelochemicals on parasitoid development. Camphell and Duffey^32,33^ had reported higher mortality rates, morphological deformities, increased development time, decreased adult weight and longevity of *Hyposoter exiguae* (Viereck) developing in caterpillars of tomato fruitworm, *Heliothis zea* (Boddie) fed on artificial diet containing α tomatine, a glycol alkaloid as compared to those fed on control diet. Rutin, a phenolic compound also exerted indirect negative effects on *H. exigua* fed on treated *H. armigera*^34^. Linear furanocoumarin too was reported to increase larval mortality of the host *S. exigua* and its parasitoid *Archytas marmoratus* (Townsend)^8^. Corroboratory results were obtained for the parasitoid, *B. hebetor* when it was allowed to parasitize *S. litura* larvae which had been fed gallic acid incorporated diet. However, it was noticed that these effects were significantly greater at higher concentration of gallic acid (LC_50_) than at lower concentration (LC_30_) when compared to control. Larval mortality of *B. hebetor* larvae increased and adult emergence decreased as the concentration of gallic acid supplemented in diet of *S. litura* larvae increased from LC_30_ to LC_50_. While the larval period and pupal period of *B. hebetor* larvae was not significantly affected, the time taken by the larvae to develop into adults also showed a concentration dependent increase. These findings indicated that toxicity of gallic acid to *S. litura* as well as its parasitoid increased with increase in concentration. Similar to our findings, El-Heneidy et al.^35^ had reported that high levels of nicotine in artificial diet of *S. frugiperda* (JE smith) lowered the survival rate of larvae of its parasitoid, *Hyposter annulipas* (Coresson), prolonged the developmental time and resulted in small sized adults. Reitz and Trumble^36^ had also observed that fewer adults of parasitoid, *Copidosonma floridarum* (Ashmead) emerged from broods developing on cabbage looper, *Trichoplusia ni* (Hubner) fed on artificial diet containing higher concentration of three furanocoumarins viz*.,* psoralen, xanthotoxin and bergapten when compared to diet having lower furanocoumarin concentrations.

Gallic acid also affected the hatching of *B. hebetor* larvae which was considerably less in *S. litura* larvae fed LC_50_ concentration than at LC_30_ concentration when compared to control. Mondy et al.^37^ had observed that nutrients obtained from host affect egg viability and hatching in parasitoids. *S. litura* larvae feeding on diet having higher levels of gallic acid may have been a poor quality host which could have been due to diversion of energy and resources to detoxify the phenolic acid or could have resulted from gallic acid toxicity. Punia et al.^38^ also reported deterrence of host quality under the influence of ellagic acid which indirectly impacted the survival of its parasitoid, *B. hebetor*. Barbosa and Saunders^39^ had reported that toxic substances in plants retard growth, reduce vigour or kill susceptible herbivores and can cause physiological or metabolic changes in parasitoids.

Increasing concentration of gallic acid provided to *S. litura* larvae decreased parasitization by *B. hebetor* adults. The eggs laid per host as well as fecundity of *B. hebetor* adults decreased in the treated larvae. However the decrease was not significant. Furanocoumarins viz*.,* isopimpinellin and xanthotoxin were also found to decrease the parasitization rate and clutch size of both male and female of polyembryonic parasitoid, *Copidosoma sosares* (Walker) when reared on hosts fed on higher concentrations of the compounds^40^.

Growth inhibitory effects of gallic acid were less severe on parasitoid when compared to positive control (azadirachtin). The toxic effect of azadirachtin on larval stages and adult emergence of *Trichogramma chilonis* Ishii was also reported by Narendra et al.^41^. Commercial formulations of azadirachtin had also been reported to negatively affect the life table parameters of *Habrobracon hebetor* (Say) by Abedi et al.^42^. Our findings showed 93.33% mortality of *S. litura* larvae at higher concentration of azadirachtin. In field conditions this may indirectly affect the survival of natural enemies as the mortality of prey population is high. This would result in reduction of food source for natural enemies and thus lead to decline in natural enemy population through starvation^43,44^.

Hemocytes form an important component of immune system in insects. Plant metabolites can impact parasitoid survival by altering the immune response of insects^45^. In the present study a significant decline in total hemocyte count and increase in hemocyte deformity was observed in *S. litura* larvae when fed on gallic acid treated diet when compared with control. Similarly, Ayyangar and Rao^46^ had also reported significant decline in total haemocyte count in the haemolymph of final instar larvae of *S. litura* when treated with azadirachtin. Zibaee and Bandani^47^ had also reported a significant dose dependent decline in total hemocyte count of adults of sunn pest, *Eurygaster integriceps* (Puton) when fed on diet containing *Artemisia annua* extract. Crude aqueous leaf extract of *C. inerme* had also resulted in lower haemocyte count in the 6th instar larvae of the cotton bollworm, *Helicoverpa armigera* (Hubner)^48^. Plumbagin, a phytochemical, was also found to drastically reduce the haemocytes of red cotton bug, *Dysdercus koenigii* (F.)^49^*.* The findings indicate that feeding on gallic acid incorporated diet compromised the cellular immune response of *S. litura* larvae and thereby increased its susceptibility to attack by the parasitoid.

Oxidation of phenolic compounds generate free radicals which can affect the growth and survival of insects^50,51^. Upregulated expression of genes for the antioxidant enzymes, SOD, Glutathione peroxidae and POX indicate their involvement in mitigating oxidative stress in the larvae generated by the ingestion of gallic acid incorporated diet. Also the expression of the genes encoding esterases and GSTs increased in *S. litura* larvae with treatment. Insects show resistance to allelochemicals via short term induction of detoxification enzymes^52^. The involvement of the GST super family in the detoxification of various plant xenobiotics has been reported by Ref.^53^. Various xenobiotics and endogenous compounds, including insecticides, drugs, insect hormones, organic solvents, allelochemicals and host plants lead to the induction of detoxification enzymes viz*.,* glutathione transferases and esterases^54^. Li et al.^55^ also reported the involvement of esterase in allelochemical metabolism.

It can be concluded from the present findings that gallic acid at lower concentrations impaired the growth of *S. litura* but only slightly affected the development of *B. hebetor*. However higher concentrations of gallic acid were toxic to both the host as well as its parasitoid. This study could provide baseline data to plant breeders for enhancing resistance traits in plants against insect pests without causing any significant harm to their parasitoids.

## Methods

The cultures of *S. litura*, *B. hebetor* and rice moth, *Corcyra cephalonica* (Stainton) were maintained in the laboratory at standard conditions of 25 ± 2 °C temperature, 65 ± 5% relative humidity (RH) and 12:12 (D:L) photoperiod.

### *Spodoptera litura* rearing

The *S. litura* were reared on fresh castor leaves, *Ricinus communis* (L.) under standard conditions in the Insect Physiology laboratory of Guru Nanak Dev University, Amritsar. The glass jars (15 cm × 10 cm) having fresh castor leaves, *R. communis* were used for culturing until pupation. On pupation, the pupae were transferred to pupation jars (15 cm × 10 cm) which had 4-5 cm of moist sand covered with filter paper. On emergence, the adults were shifted to oviposition jars (15 cm × 10 cm) lined with filter paper to facilitate egg laying, secured with muslin cloth to check their escape and provided with cotton swab soaked with water and honey solution (4:1) to serve as food.

### *Bracon hebetor* rearing

The parasitoid, *B. hebetor* was reared on 5th instar larvae of *C. cephalonica*. The culture of *C. cephalonica* was reared on partially crushed sorghum grains at standard conditions of temperature (25 ± 2 °C) and humidity (65 ± 5% RH). The freshly emerged adult parasitoids in ratio 1:2 (male:female) were transferred to glass chimneys having a cotton swab soaked in water and honey (4:1) solution to serve as food. The parasitoid, *B. hebetor* was provided with healthy 5th instar larvae of *C. cephalonica* for parasitization and parasitized larvae were observed for egg laying under microscope and were then kept inside tissue papers in plastic petri plates (90 mm × 15 mm) until cocoon formation. The cocoons formed were shifted into sterile solo cups (4 cm × 6 cm) and the adults emerged were allowed to mate for 24 h. These newly emerged *B. hebetor* larvae were then used for bioassay studies.

### Chemicals used

Gallic acid and azadirachtin with 95% purity were obtained from Sigma Aldrich Pvt. Ltd., India.

### Conduction of experiment

#### Bioassays with *S. litura*

To evaluate the effect of gallic acid on *S. litura* and its parasitoid, stock solution having concentration 15625 ppm was prepared in distilled water and added in artificial diet of the insect in amounts required to have the desired concentrations i.e. 5, 25, 125, 625 and 3125 ppm. Diet without gallic acid was taken as control. These concentrations were delivered to the insect when it fed on the diet. Azadirachtin incorporated diet with different concentrations i.e. 5, 25, 125, 625 and 3125 ppm was taken as positive control. The antibiosis effect of gallic acid on *S. litura* was ascertained by feeding the six days old larvae on diet supplemented with 5, 25, 125, 625 and 3125 ppm of the compound. The larvae were kept in sterilized plastic containers containing treated and control diet and were examined daily for different parameters viz*.,* the larval period, total development period, larval mortality, pupal weight and adult emergence. The LC_30_ and LC_50_ concentrations were ascertained from the data obtained. The experiments had six replicates with five larvae in each replicate and the experiments were repeated twice.

#### Nutritional assays with *S. litura*

A 3 days experiment was conducted with 6 days old larvae of *S. litura* according to Koul et al.^56^ using different concentrations of gallic acid and azadirachtin (5 ppm, 25 ppm, 125 ppm, 625 ppm and 3125 ppm) along with control. The larvae were weighed and released into sterilized plastic container already containing weighed treated and control diet. The larvae were allowed to feed for 72 h and then annotations were made for final larval weight, diet left and fecal matter (in mg) and the dry weights for the same were taken after incubating for 72 h at 60 °C inside an incubator. The dry weight readings served the purpose of water loss under controlled conditions. From this data, the following nutritional indices were calculated on dry weight basis after 3 days of feeding as proposed by Waldbauer^57^. Each concentration including control was replicated 6 times with 5 larvae in each replicate and the experiment was repeated twice.

$$ {\text{RGR}} = \frac{{{\text{Change}}\,{\text{in}}\,{\text{larval}}\,{\text{dry}}\,{\text{weight/day}}}}{{{\text{Initial}}\,{\text{larval}}\,{\text{dry}}\,{\text{weight}}}} $$

$$ {\text{RCR}} = \frac{{{\text{Change}}\,{\text{in}}\,{\text{diet}}\,{\text{dry}}\,{\text{weight/day}}}}{{{\text{Initial}}\,{\text{larval}}\,{\text{dry}}\,{\text{weight}}}} $$

$$ ECI \, = \frac{{{\text{Dry}}\,{\text{weight}}\,{\text{gain}}\,{\text{of}}\,{\text{insect}}}}{{{\text{Dry}}\,{\text{weight}}\,{\text{of}}\,{\text{food}}\,{\text{ingested}}}} \times 100 $$

$$ {\text{ECD}} = \frac{{{\text{Dry}}\,{\text{weight}}\,{\text{gain}}\,{\text{of}}\,{\text{insect}}}}{{{\text{Dry}}\,{\text{weight}}\,{\text{of}}\,{\text{food}}\,{\text{ingested}} - {\text{Dry}}\,{\text{weight}}\,{\text{of}}\,{\text{frass}}}} \times 100 $$

$$ {\text{AD}} = \frac{{{\text{Dry}}\,{\text{weight}}\,{\text{gain}}\,{\text{of}}\,{\text{insect}} - {\text{Dry}}\,{\text{weight}}\,{\text{of}}\,{\text{frass}}}}{{{\text{Dry}}\,{\text{weight}}\,{\text{of}}\,{\text{food}}\,{\text{ingested}}}} \times 100 $$

where RGR = Relative growth rate, RCR = Relative consumption rate, ECI = Efficiency of conversion of ingested food, ECD = Efficiency of conversion of digested food, AD = Approximate digestibility.

### Bioassays with *B. hebetor*

The effect of gallic acid and azadirachtin on parasitization and fecundity of *B. hebetor* was studied by allowing the newly emerged adults to mate for 24 h and the mated adults were then transferred to glass chimney in the ratio female: male (2:1). The rearing of *S. litura* larvae upto 3rd instar stage was done on diet amended with LC_30_ and LC_50_ concentrations of gallic acid as well as on control (unamended diet). A single treated 12 days old *S. litura* larvae was then exposed to parasitoid wasp for 24 h in the chimney and then the parasitized larvae were transferred on tissue paper in the petriplates (90 mm × 14 mm). The paralyzed larvae were observed daily under stereo microscope (Magnus) at 40X magnification to observe parasitoid eggs for various parameters. In this way, *S. litura* larvae fed on different concentrations of gallic acid in artificial diet were exposed daily till the female died in each treatment.

### Effect of gallic acid and azadirachtin on the development of *B. hebetor*

The effect of gallic acid and azadirachtin on development of *B. hebetor* was evaluated by taking two days old mated females in glass chimneys (two wasps per chimney). The females were allowed to oviposit on the larvae of *S. litura* (3rd instar) reared on artificial diet amended with LC_30_ and LC_50_ concentration of gallic acid. The treated larvae of *S. litura* were provided to parasitoid wasps for 24 h. The parasitized *S. litura* larvae were removed from the chimneys and fresh hosts were provided to the wasps daily. The larvae removed from the chimney were placed individually in petri plates (90 mm × 14 mm) and were allowed to complete development. The petri plates were checked daily for development of parasitoid larvae. Cocoons of the parasitoid were also checked daily and the time of emergence of wasps was recorded. On emergence, the number and sex ratio of the progeny were noted. The experiment was replicated six times with two females per replicate and a total of 60 larvae were exposed to parasitoid wasps for each treatment and control.

### Cellular immune response of *S. litura*

To evaluate the effect of gallic acid on immune response of *S. litura*, 3rd instar larvae (12 days old) were fed on artificial diet supplemented with LC_30_ and LC_50_ concentrations for different time intervals i.e. 24, 48, 72, 96 and 120 h. The treated larvae along with the larvae fed on control diet were kept at standard conditions of temperature and humidity i.e. 25 ± 2 °C and 65 ± 5%, respectively. The hemolymph was collected by piercing the prothoracic legs with a sterile needle. For each time interval, ten larvae were randomly selected from each treatment group and the hemolymph was collected. The pooled hemolymph was used to study the total haemocyte count (THC) and deformities in haemocytes according to the protocol of Tauber and Yeager^58^ and Arnold and Hinks^59^, respectively. All experiments were replicated twice.

### Gene expression of antioxidant enzymes

The relative expression of genes related with different antioxidant and detoxifying enzymes was measured using quantitative RT-PCR in the larvae of *S. litura* treated with the LC_50_ concentration of gallic acid at different intervals (48 and 72hours). The total RNA was extracted using Trizol method (Invitrogen). The quality and concentration of total RNA was checked using agarose gel electrophoresis (1%) and nanodrop spectrophotometer. The iScript cDNA synthesis kit of Biorad was used to synthesize cDNA from 1.0 μg of total RNA. The cDNA obtained was stored at − 20 °C for further use after a dilution of tenfold. The mRNA sequences of various genes used to design primers of genes of interest were obtained from NCBI and actin was used as an internal reference gene (Table 9). Power SYBR Green PCR Master Mix (Applied Biosystems) with Step One Real Time PCR System (Applied Biosystems) was used to perform the qRT-PCR for different genes. The relative gene expression was determined by using the 2^−ΔΔCT^ method to calculate the threshold values (Ct)^60^.

**Table 9:** Primer sequences used for gene expression analysis using qRT-PCR.

Gene name	Primer sequence	Annealing temperature	Product size	GenBank accession number
Actin	Forward primer 5′TGCGTGACATCAAGGAGAAG 3′	52	191	XM_022975383.1
	Reverse Primer 5′GCAAGCTTCCATACCCAAGA3′	52		
SOD	Forward primer 5′GGCAAAGGGGCTACATGTCT3′	54	188	XM_022958483.1
	Reverse primer 5′ATACGTTTCCGAGGTCACCG3′	54		
EST	Forward primer 5′TGCAATGCTTTGGGCAACTAA3′	50	191	XM_022972575.1
	Reverse primer 5′TCGCTTTTGATTCATCTGGTTGTC3′	54		
GST	Forward primer 5′TTACTTTGGAGGGCATCGTC3′	50	190	KF482978.1
	Reverse primer 5′TTGTGGTGAGTTCGCATGTT3′	50		
POX	Forward primer 5′TACTGACGGTCATGCACACT3′	53	229	XM_022970394.1
	Reverse primer 5′CCGTCCCAGTAACCCTCTTT3′	53		
GPOX	Forward primer 5′AAAGCTGCGACATCCATCCA3′	52	175	XM_022972377.1
	Reverse primer 5′TGTCCTCGGCATATTTCTCGT3′	52		

### Statistical analysis

The data were represented as their means ± SE. The one-way analysis of variance (ANOVA) along with Tukey’s test at *p* ≤ 0.05 was done to check the differences in means. SPSS software for windows version 16.0 (SPSS Inc, Chicago), Microsoft office Excel 2007 (Microsoft Corp., USA) and ASSISTAT software were used to perform the statistical analysis. The LC_30_ and LC_50_ values were calculated by applying Probit analysis using SPSS software version 16.0 for windows.

### Ethical approval

This article does not contain any studies with human participants or animals performed by any of the authors.
